# Heterologous Expression of CLIBASIA_03915/CLIBASIA_04250 by *Tobacco Mosaic Virus* Resulted in Phloem Necrosis in the Senescent Leaves of *Nicotiana benthamiana*

**DOI:** 10.3390/ijms21041414

**Published:** 2020-02-19

**Authors:** Hui Li, Xiaobao Ying, Lina Shang, Bryce Redfern, Nicholas Kypraios, Xuejun Xie, FeiFei Xu, Shaopeng Wang, Jinghua Zhang, Hongju Jian, Hongtao Yu, Dianqiu Lv

**Affiliations:** 1 College of Agronomy and Biotechnology, Southwest University, Chongqing 400715, China; lihui921137@swu.edu.cn (H.L.); sln2019@swu.edu.cn (L.S.); hjjian25@swu.edu.cn (H.J.); 2 State Cultivation Base of Crop Stress Biology for Southern Mountainous Land of Southwest University, Chongqing 400715, China; 3 Gulf Coast Research and Education Center, University of Florida, Wimauma, FL 33598, USA; yingxb75@ufl.edu (X.Y.); killerblr@ufl.edu (B.R.); nkypraios@ufl.edu (N.K.); 4 Changzhou Institute of Technology, Changzhou 213032, China; xiexj@czust.edu.cn; 5 Heilongjiang Academy of Agricultural Sciences, Harbin 10086, Chinawangshaopeng2010@126.com (S.W.); zhangjinghua010@hotmail.com (J.Z.); yuhongtao@163.com (H.Y.)

**Keywords:** Huanglongbing, virulence factor, phloem necrosis, subcellular localization, protein–protein interaction

## Abstract

Huanglongbing (HLB), also known as citrus greening, is the most notorious citrus disease worldwide. *Candidatus* Liberibacter asiaticus (*Ca*Las) is a phloem-restricted bacterium associated with HLB. Because there is no mutant library available, the pathogenesis of *Ca*Las is obscure. In this study, we employed *tobacco mosaic virus (*TMV) to express two mature secretion proteins CLIBASIA_03915 (m03915) and CLIBASIA_04250 (m04250) in *Nicotiana benthamiana* (*N. benthamiana*). Phloem necrosis was observed in the senescent leaves of *N. benthamiana* that expressed the two low molecular weight proteins, while no phloem necrosis was observed in the plants that expressed the control, green fluorescent protein (GFP). Additionally, no phloem necrosis was observed in the senescent leaves of *N. benthamiana* that expressed the null mutation of m03915 and frameshifting m04250. The subcellular localizations of m03915 and m04250 were determined by fusion with GFP using confocal microscopy. The subcellular localization of m03915 was found to be as free GFP without a nuclear localization sequence (NLS). However, m04250 did have an NLS. Yeast two-hybrid (Y2H) was carried out to probe the citrus proteins interacting with m03915 and m04250. Six citrus proteins were found to interact with m03915. The identified proteins were involved in the metabolism of compounds, transcription, response to abiotic stress, ubiquitin-mediated protein degradation, etc. The prey of m04250 was involved in the processing of specific pre-mRNAs. Identification of new virulence factors of *Ca*Las will give insight into the pathogenesis of *Ca*Las, and therefore, it will eventually help develop the HLB-resistant citrus.

## 1. Introduction

Huanglongbing (HLB), also known as citrus greening, is the most notorious citrus disease worldwide. The typical symptoms of HLB include yellowing of the veins, dieback of twigs, decline of roots, fruits unable to develop proper color, and, eventually, the death of the infected trees [1,2]. HLB is associated with a phloem-restricted Gram-negative bacterium, named *Candidatus* Liberibacter africanus (*Ca*Laf), *Ca*. Liberibacter asiaticus (*Ca*Las), and *Ca*. L. americanus (*Ca*Lam) based on geographic distribution. *Ca*Las is the most virulent pathogen with global distribution [1]. *Ca*Las caused a 72.2% reduction in orange juice and a 20.5% reduction in the fresh fruit market in the United States from 2007–2008 to 2017–2018, respectively [3]. All commercial citrus cultivars are susceptible to HLB, and there is no cure for HLB [2]. Therefore, effective management of HLB is urgently needed. However, the lack of in-depth knowledge of the virulence mechanism of *Ca*Las prevents the development of effective HLB management.

Though *Ca*Las remains uncultured, the availability of *Ca*Las genomic information [4,5] facilitates research on the virulence mechanism of *Ca*Las. *Ca*Las lacks type III, and type IV secretion systems (TIIISS, TIVSS) to deliver effectors into host cells [4]. The effectors of *Ca*Las mainly rely on Sec secretion machinery to enter host cells. Several reports have used transient expression or viral vectors to identify the effectors of *Ca*Las [6,7,8]. *Ca*L5315 was reported as a lethal effector, causing cell death in infiltrated *N. benthamiana* leaves by transient expression [6]. CLIBASIA_00460 caused necrosis in the systemic leaves when expressed by *Potato virus X* (PVX) in *N. benthamiana* [7]. CLIBASIA_00470 and CLIBASIA_04025 were reported to inhibit *N. benthamiana* growth when expressed by *tobacco mosaic virus* (TMV) at two weeks post infiltration (WPI) [8]. 

Several reports have shown that *Ca*Las encodes proteins to suppress citrus disease resistance as the virulence mechanism [9,10,11]. *SC2_gp095* encodes a peroxidase that downregulates *RbohB* when expressed transiently in plants [10]. *Ca*Las also encodes a salicylic acid (SA) hydroxylase to downregulate the accumulation of SA in plants, therefore, inhibiting citrus disease resistance. *Ca*Las05315, also called Sec-delivered effector 1 (SDE1), was found to interact with citrus papain-like cysteine proteases (PLCPs) and inhibited their activities. PLCPs confer plant disease resistance. SDE1 transgenic citrus exhibited the suppression of PLCPs [11]. The inhibition of defense response is one of the strategies of *Ca*Las to establish colonization in citrus. *Ca*Las caused phloem anatomical changes such as phloem necrosis, phloem plugging, and phloem collapse [12]. The phloem of the flowering plant consists of companion cells and mature sieve elements to transport phloem sap from source to sink [13]. Young sieve elements lose their ribosomes, dictyosomes, nucleus, and cytoskeleton during the transition to mature sieve elements [14]. The mature sieve elements contain smooth endoplasmic reticulum (ER), mitochondria, sieve element plastids, and phloem proteins [15]. It was recently reported that *Ca*Las encodes proteins to interact with the key proteins of lignin production [8], which is the main component of woody cell wall biomass (up to 30%) [16]. 

We reported two ultra-low molecular weight putative virulence factors CLIBASIA_03915 (m03915) and CLIBASIA_04250 (m04250) that caused phloem necrosis in senescent leaves of *N. benthamiana* when expressing the mature proteins (m03915 and m04250) by TMV. The two hypothetical proteins were Sec-dependent secretory proteins confirmed by phoA assay. The two putative virulence factors were confirmed by mutation assay to function at the protein level. Green fluorescence protein (GFP) was used to tag m03915 and m04250 to determine their subcellular localizations. It was found that m04250 localized in the nucleolus. Yeast two-hybrid (Y2H) was applied to identify host proteins that interacted with the two effectors. The putative mechanisms of phloem necrosis caused by m03915 and m04250 and the potential application of our findings in citrus improvement are discussed.

## 2. Results

### 2.1. CLIBASIA_03915 and CLIBASIA_04250 Were Sec-Dependent Proteins

**Figure 1:**
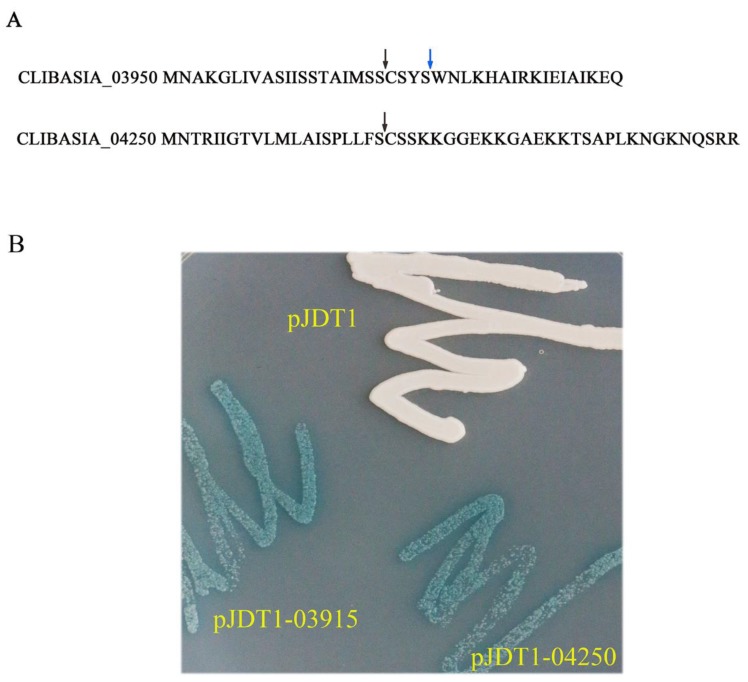
Prediction and confirmation of signal peptides of CLIBASIA_03915 and CLIBASIA_04250. (**A**) The signal peptides of CLIBASIA_03915 and CLIBASIA_04250 were predicted by SingalP 3.0 and SingalP 5.0. The cleavage site of CLIBASIA_03915 was between 20–21 aa (SignalP 5.0) or 24-25 aa (SignalP 3.0). SignalP 3.0 and SignalP 5.0 predicted the same cleavage site of CLIBASIA_04250 (20-21 aa). The arrows indicated the cleavage sites. (**B**) The signal peptides of CLIBASIA_03915 and CLIBASIA_04250 were confirmed by phoA assay. The *E*. *coli* cells harboring the recombinant plasmids along with the negative control pJDT1 were streaked on Luria–Bertani (LB) plates amended with 5-bromo-4-chloro-3-indolylphosphate (BCIP) and sodium phosphate. The negative control pJDT1 did not cause the color change. The *E*. *coli* cells expressing the recombinant plasmids turned to blue.

The signal peptides of CLIBASIA_03915 and CLIBASIA_04250 were predicted by SignalP 3.0 and SignalP 5.0. The cleavage site of CLIBASIA_03915 was between 24-25 aa (SignalP 3.0) or 20-21 aa (SignalP 5.0), and the cleavage site of CLIBASIA_04250 was between 20-21 aa (SignalP 3.0 and SignalP 5.0). The full-length DNA fragments of CLIBASIA_03915 and CLIBASIA_04250 with added enzyme sites were introduced into vector pJDT1, which contained the *phoA* gene (alkaline phosphatase) lacking the signal sequence [17] to have pJDT1-03915 and pJDT1-04250. The *Escherichia coli* clones harboring the positive recombinant plasmids along with the control pJDT1were streaked on Luria–Bertani (LB) plates amended with 5-bromo-4-chloro-3-indolylphosphate (BCIP) and sodium phosphate. The cells expressing pJDT1-03915 and pJDT1-04250 turned blue while the cells expressing the negative control (pJDT1) remained unchanged in color (Figure 1). This finding indicated that CLIBASIA_03915 and CLIBASIA_04250 were Sec-Dependent proteins.

### 2.2. Heterologous Expression of the Mature Proteins of CLIBASIA_03915 (m03915) and CLIBASIA_04250 (m04250) Caused Phloem Necrosis in the Senescent Leaves of N. benthamiana

**Figure 2:**
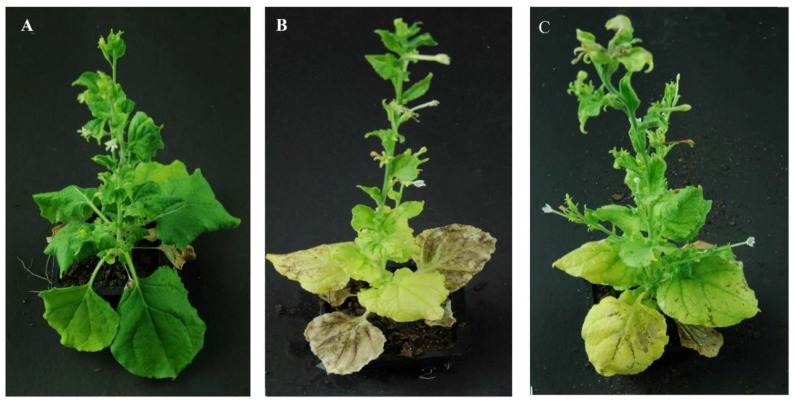
Mature proteins of CLIBASIA_03915 (m03915) and CLIBASIA_04250 (m04250) caused the phloem necrosis in the senescent leaves of *Nicotiana benthamiana*. Plants expressing control green fluorescent protein (GFP) did not show phloem necrosis (**A**). On the contrary, expression of m03915 (**B**) and m04250 (**C**) by tobacco mosaic virus in *N. benthamiana* plants caused phloem necrosis in senescent leaves. The pictures were taken at 5 weeks post infiltration (WPI).

TMV was used to express virulence factors of *Ca*Las and resulted in identifying two virulence factors that inhibited plant growth at two weeks post infiltration (WPI) [8]. Heterologous expression of m03915 and m04250 in *N. benthamiana* by TMV showed a similar phenotype to control GFP at 2 WPI. However, phloem necrosis was observed in the senescent leaves of plants that expressed m03915 and m04250 at 5 WPI (Figure 2B,C). No phloem necrosis was observed in the senescent leaves of *N. benthamiana* that expressed GFP at the same time (Figure 2A).

### 2.3. The Mutated m03915 and m04250 Could Not Cause Phloem Necrosis in the Senescent Leaves

**Figure 3:**
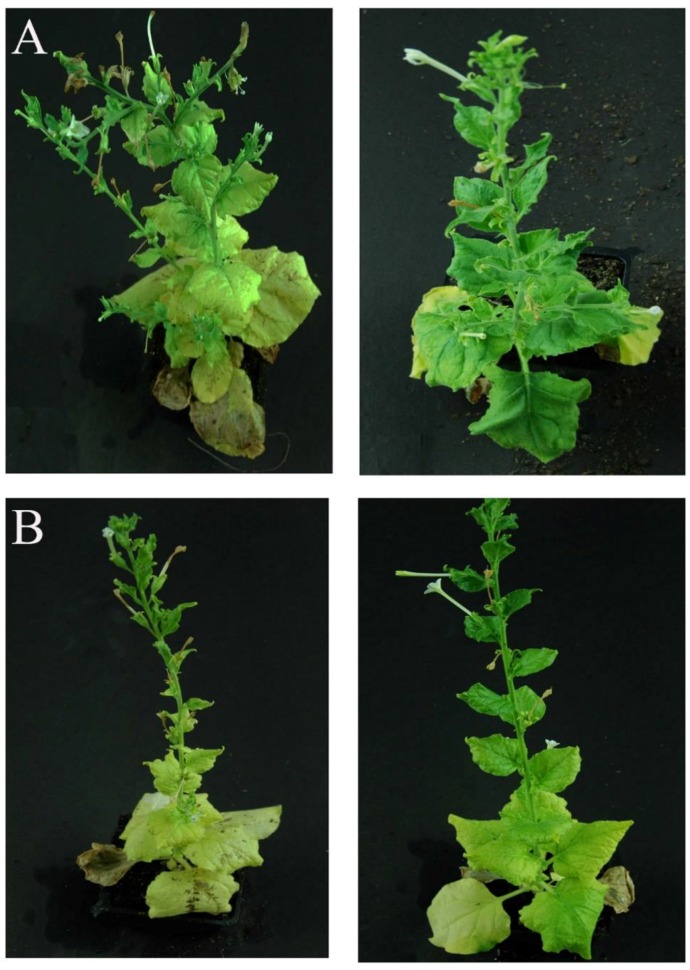
The mutated m03915 (m03915Nu, on right) and m04250 (m04250FS, on right) could not cause phloem necrosis, while m03915 (on left) and m04250 (on left) caused phloem necrosis at 5 WPI. (**A**) The mutated m03915 (m03915Nu, on right) could not cause phloem necrosis, while m03915 (on left) caused phloem necrosis at 5 WPI. (**B**) The mutated m04250 (m04250FS, on right) did not cause phloem necrosis, while m04250 (on left) caused phloem necrosis at 5 WPI.

To exclude the possibility that phloem necrosis was caused by virus-induced gene silencing (VIGS), mutations were introduced into m03915 and m04250. The null mutation was introduced into m03915 by replacing the start codon ATG with the stop codon TAA to generate the mutant m03915Nu. The frameshifting mutation was introduced into m04250. The frameshifting mutation of m04250FS led to early termination of translation (after two amino acids FF). At 5 WPI, m03915 caused phloem necrosis in the senescent leaves, while no phloem necrosis was observed in the senescent leaves of *N. benthamiana* expressing m03915Nu (Figure 3A). Plants expressing m04250FS did not show the phenotype of phloem necrosis as native m04250 (Figure 3B). The results indicated that m03915 and m04250 functioned at the protein level rather than at the RNA level to cause phloem necrosis.

### 2.4. Determination of the Subcellular Localizations of m03915 and m04250

**Figure 4:**
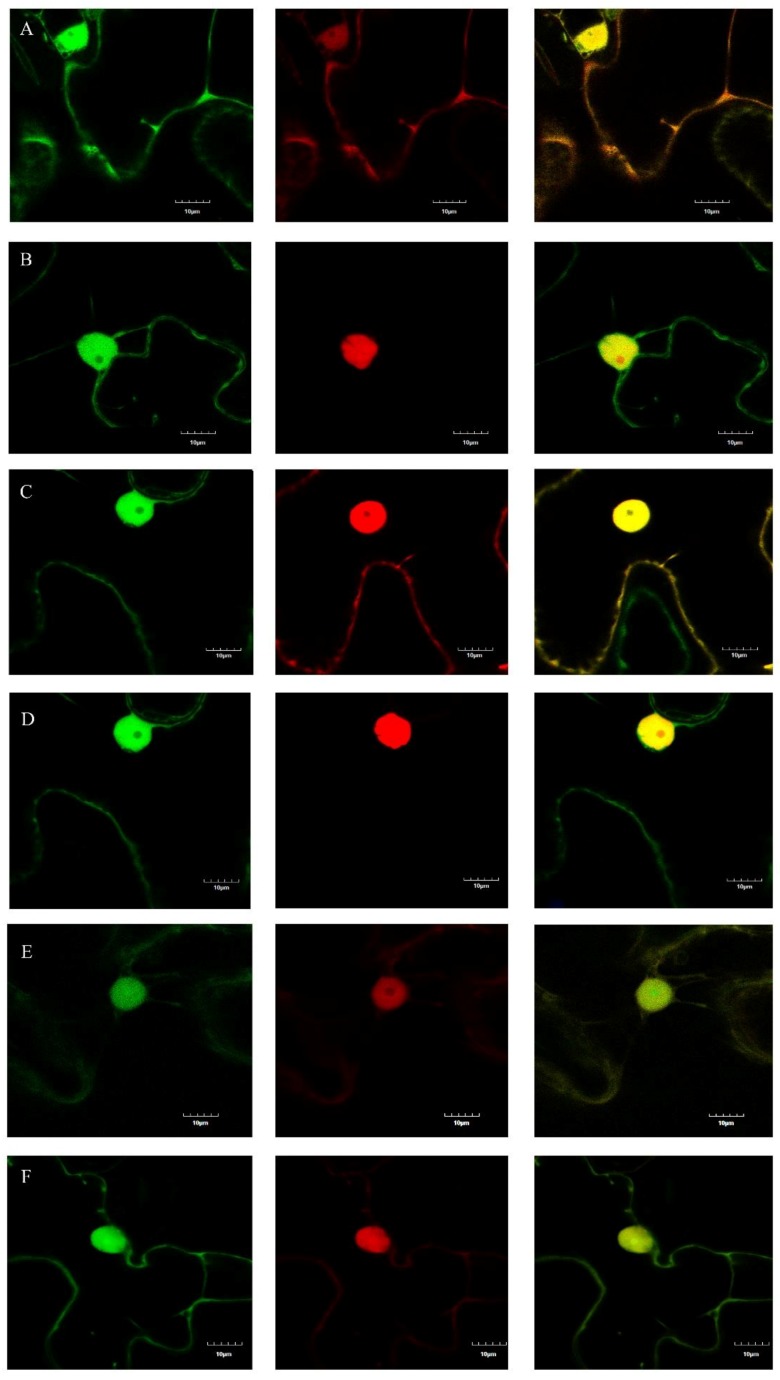
Determination of subcellular localizations of m03915 and m04250. GFP was used to determine the subcellular localizations of m03915 and m04250 by fusion at C-terminal of m03915 and m04250 using confocal microscopy. Free GFP was used as a control. Six true leaf-stage *N. benthamiana* were infiltrated with *Agrobacterium* EHA 105 harboring the desired plasmid. Red fluorescent protein and red fluorescent protein with nuclear localization signal (NLS) (RFP-NLS) were used for coinfiltration to determine whether m03915 and m04250 had an NLS. The samples were collected at two days post infiltration and observed under a confocal microscope. (**A**) free GFP coinfiltrated with RFP; (**B**) free GFP coinfiltrated with RFP-NLS; (**C**) m03915-GFP coinfiltrated with RFP; (**D**) m03915-GFP coinfiltrated with RFP-NLS; (**E**) m04250-GFP coinfiltrated with RFP; (**F**) m04250-GFP infiltrated with RFP-NLS. The left images showed the GFP channel, the middle images showed the RFP channel; the right images showed the overlay of GFP and RFP. GFP and m03915-GFP did not have an NLS. On the contrary, m04250-GFP localized in the nucleus.

GFP was used to detect the subcellular localizations of m03915 and m04250. The patterns of distribution of free GFP, m03915-GFP, and m04250-GFP were similar in *N. benthamiana* cells (Figure S1). Red fluorescent protein (RFP) and RFP-nuclear localization signal (NLS) were co-expressed with GFP/m03915-GFP/m04250-GFP in *N. benthamiana* to determine whether an NLS was in m03915-GFP or m04250-GFP. Free GFP and free RFP were found to be distributed in the cytosol and nuclear lumen (Figure 4A). RFP was observed in the cell nucleus when containing an NLS. Free GFP could not enter the nucleolus (Figure 4B). The subcellular localization of m03915 was the same as free GFP, no NLS was present (Figure 4C,D). However, m04250-GFP showed stronger fluorescence in the nucleolus (Figure 4E,F). This indicated that m04250 localized in multiple cellular compartments, including the nucleolus.

### 2.5. Identification of the Host Proteins Interacting with m03915 and m04250

Yeast two-hybrid (Y2H) was applied to identify citrus proteins interacting with m03915 and m04250. The second Glutamic acid of m03915 was changed to Alanine (m03915E20A) to eliminate the autoactivation activity. Six genes (seven isolates) were identified from the library that interacted with m03915E20A. XM_006465843 was annotated as a cytochrome P450 71A1-like protein. XM_006467727.3 encoded the transcription factor bHLH16. The annotation of XM_006478039.3 was a glutamate decarboxylase 5-like protein. XM_006452415 encoded a cold and drought-regulated protein. Interestingly, XM_006481033 was annotated as an ubiquitin-activating enzyme E1 1-like protein. Two isolates were annotated as an EG45-like domain-containing protein (accession number XM_006474049). One isolate which interacted with m04250 was annotated as a suppressor of mec-8 and unc-52 protein homolog 2 protein (access number XM_006474512) (Table 1).

## 3. Discussion

*Ca*Las is a phloem restricted uncultured Gram-negative bacterium. It is directly delivered into citrus phloem by the vector of Asian citrus psyllid (ACP) under natural conditions [18]. The phloem sap of pineapple sweet orange contains 20 amino acids, 8 sugars, and 7 organic acids as nutrition for *Ca*Las and psyllid [19]. ACP delivers *Ca*Las directly into citrus phloem to avoid triggering citrus immunity responses. Moreover, *Ca*Las suppresses plant disease resistance by the degradation of defense signals [9,10], such as SA and H_2_O_2_, which participate in local hypersensitivity reactions as well as systemic acquired resistance [20,21]. Efforts have been made to understand the HLB-tolerance mechanism of citrus [22,23,24,25,26,27] and how HLB symptoms develop in citrus [28,29,30]. However, it is still unclear how citrus develops HLB symptoms upon *Ca*Las infection, including phloem necrosis.

Based on the genomic information of *Ca*Las, several reports focused on the virulence mechanisms of *Ca*Las [7,8,9,10]. The identified effectors could cause cell death of infiltrated leaves [6,8], systemic leaves necrosis [7], and inhibition of plant growth [8]. Mature proteins of CLIBASIA_03915 (m03915) and CLIBASIA_04250 (m04250) caused phloem necrosis in the senescent leaves of *N. benthamiana* when expressed by TMV (Figure 2) and functioned at the protein level (Figure 3). Six proteins were identified to interact with m03915E20A (Table 1). Cytochrome P450s play critical roles in the synthesis and metabolism of lignin, sterols, isoflavonoids, terpenes, flavonoids, furanocoumarins, and other secondary plant products [31]. The expression of cytochrome P450 was upregulated in the HLB tolerant citrus under biotic and abiotic stress [25]. Genes involved in cytochrome P450-related reactions were upregulated in HLB tolerant citrus [32]. Basic helix–loop–helix (bHLH) transcription factors are the second largest group of transcription factors in plants that are also involved in plant development, phytohormone signaling, and stress responses [33]. It was reported that AtbHLH162 was involved in the response to fungal pathogen [34]. Glutamate decarboxylase 5 catalyzes the production of γ-aminobutyric acid (GABA) [35]. The concentration of GABA was higher in *Ca*Las infected ACP than in the control, and therefore, GABA might be from HLB infected citrus [36]. XM_006452415 encoded a cold and drought-regulated protein by annotation. However, the proteins that hit XM_006452415 can be grouped into cold and drought-related protein, phase-change related protein, glycine-rich protein, glycine-rich cell wall structural protein-like, etc. The exact function of XM_006452415 needs to be determined. Interestingly, a protein annotated as a ubiquitin-activating enzyme E1 1-like protein (UBA1) was found to interact with m03915E20A. The ubiquitin–proteasome system regulates plant development, flowering, responses to abiotic stresses, and biotic stresses [37]. UBA1 was required for disease resistance in Arabidopsis [38]. Plant pathogens target the key enzymes in the ubiquitin-conjugating cascade to suppress plant disease resistance [39]. It was reported that CLIBASIA_00470 inhibited plant growth and interacted with polyubiquitin-like proteins and a putative E3 protein [8]. It is possible that m03915 downregulated genes in cell wall synthesis to cause phloem necrosis via ubiquitin–mediated protein degradation system. *Ca*Las may hitchhike the host ubiquitin–proteasome system to suppress citrus disease resistance and interfere with citrus development. EG45-like domain-containing protein may play a role in water and solute homeostasis [40]. Only one prey was identified to interact with m04250. It was annotated as a suppressor of mec-8 and unc-52 protein homolog 2 protein, also known as SMU2. SMU2 regulates plant pre-mRNA splicing cooperatively with SMU1 to regulate plant development [41]. The alternative splicing of genes varied significantly between HLB tolerant citrus cultivar and HLB-susceptible citrus cultivar when infected with HLB [25]. The mature protein m04250 may disturb the normal pre-mRNA splicing to cause phloem necrosis.

Since conventional breeding cannot create HLB-tolerance/resistance citrus, the transgenic approach has been used to enhance citrus HLB tolerance. The approach of overexpression has been applied to improve citrus HLB resistance [42,43,44]. However, clustered regularly interspaced short palindromic repeats (CRISPR) and CRISPR-associated proteins (CRISPR/Cas) cannot be used in citrus HLB improvement without known HLB susceptible gene [45] in spite of the advantages [46,47] and successful generation of plants that were resistant to bacterial pathogens and fungal pathogens [48] including citrus canker [49,50] by targeting *LOB1* which is the susceptibility gene of citrus canker [51]. Meanwhile, CRISPR is a powerful weapon to combat viruses [52]. However, CRISPR is only a promising tool when the virulence mechanism of *Ca*Las is still vague [45,53]. Based on our results and previous reports, we propose that E3 enzymes differential expressed in HLB tolerant and HLB susceptible citrus upon *Ca*Las infection [25] may be considered as the target of CRISPR based on the possibility of *Ca*Las interference with citrus development and disease resistance via targeting the specific proteins of the ubiquitin–proteasome system. Overexpression of the key genes in cell wall synthesis may be another way to enhance citrus HLB tolerance.

## 4. Materials and methods

### 4.1. Plant Growth Conditions

*N. benthamiana* was grown in a greenhouse at 25 °C with 16 h light and 8 h dark.

### 4.2. Prediction and Confirmation of CLIBASIA_03915 and CLIBASIA_04250 Signal Peptides

Signal peptides of CLIBASIA_03915 and CLIBASIA_04250 were predicted by combination of SignalP 3.0 [54], SignalP 5.0 [55], Phobius [56], and TMHMM [57]. To confirm the signal peptides of CLIBASIA_03915 and CLIBASIA_04250, phoA assay was employed. Vector pJDT1 [17] without the signal peptide sequence of *phoA* was used. The DNA fragments of CLIBASIA_03915 and CLIBASIA_04250 containing signal peptides were amplified with primers CLIBASIA_03915FF/CLIBASIA_03915FR, CLIBASIA_04250FF/CLIBASIA_04250FR, and respectively cloned into pGEM-T Easy vector (Promega, Madison, WI, USA, Cat. No. A1360). The correct plasmids were digested with *Sac*I and *Mfe*I, and the recovered DNA fragment was inserted into vector pJDT1. The plasmids were confirmed by sequencing, then were transformed into *Escherichia coli* (*E. coli*) strain BL21 DE3 (NEB, cat. C2527I). The *E. coli* single clone harboring CLIBASIA_03915/ CLIBASIA_04250 was streaked along with negative control pJDT1 on Luria–Bertani (LB) plates amended with 5-bromo-4-chloro-3-indolylphosphate (BCIP) (90 mg/L) and sodium phosphate (75 mM, pH 7.2).

### 4.3. Heterologous Expression of the Mature Proteins of CLIBASIA_03915 (m03915) and CLIBASIA_04250 (m04250) in N. benthamiana

*N. benthamiana* was used to express m03915 and m04250 by TMV via agroinfiltration, as described previously [8]. Primers m03915 EF/m03915 ER and m04250 EF/m04250 ER were used to amplify the DNA fragments from HLB-infected citrus without the predicted SP (SP of 03915 1-24 aa, SP of 04250 1-20 aa). Primers m03915 NF/ m03915 ER and m04250 NF/ m04250 ER were used to introduce mutations into m03915 (m03915Nu) and m04250 (m04250FS). *Agrobacterium* strain EHA105 was used to express *m03915*, *m04250*, *m03915Nu*, and *m04250FS* in *N. benthamiana* by TMV. The six-leaf stage *N. benthamiana* was used for agroinfiltration. The *agrobacterium* culture was adjusted to optical density (OD) value 0.6 by solution with 10 mM MgCl_2_, 10 mM MES pH 5.6, and 10 µM acetosyringone [58].

### 4.4. Determination of the Subcellular Localizations of m03915 and m04250

Transient expression was employed to determine the subcellular localizations of m03915 and m04250. Primers m03915 GF/m03915 GR and m04250 GF/m04250 GR were used to amplify DNA fragments to fuse with the green fluorescent protein (GFP). *Xba*I and *Xma*I sites were added at the 5′ and 3′ end of the DNA fragments, respectively. The GFP DNA fragment was amplified from plasmid p30B-GFPC3 [59] with primers GFP FF/GFP FR to introduce enzyme sites of *Xma*I and *Sac*I. The amplified DNA fragments were cloned into the pGEM-T Easy vector. The plasmids were digested with *Xba*I and *Sac*I, then the recovered DNA fragments were inserted into pBI121 [60] to get the final vectors for determination of the subcellular localizations of m03915 and m04250. The control GFP DNA fragment was amplified with primers GFP F/GFP R and inserted into pBI121. *Agrobacterium* EHA105 carrying free GFP, m03915-GFP, and m04250-GFP were cultured and OD was adjusted to 0.8 for agroinfiltration. To determine whether m03915-GFP and m04250-GFP had an NLS, red fluorescent protein (RFP) and RFP with a nuclear localization signal (NLS) were used. Plasmid pCAMBIA1380 was used as the backbone to express RFP and RFP-NLS. The DNA fragment from pBI121 between *Hin*dIII and *Eco*RI with a CaMV 35S promoter-GUS-NOS terminator was inserted into pCAMBIA1380 to get pCAMBIA1380-GUS. To eliminate the leaking expression of RFP, an intron was added at 5′ of the RFP and RFP-NLS. The amplified intron was from pBI121-GUS-INTRON with primers HAint F/ HAint R. The DNA fragment containing the intron was inserted into pCAMBIA1380-GUS between *Xba*I and *Sac*I sites to get pCAMBIA1380-INT. RFP was amplified from plasmid pSAT6-RFP-C1 [61] with primers RFP F/RFP R. The DNA fragment was recovered and inserted into pCAMBIA1380-INT between *Bam*HI and *Sac*I sites. RFP-NLS was amplified from pSAT6-mRFP-VirD2NLS [62] with primers RFP-NLS F/RFP-NLS R. The DNA fragment with RFP-NLS was inserted into pCAMBIA1380-INT between *Bam*HI and *Sac*I sites. *Agrobacterium* EHA105 carrying RFP or RFP-NLS was coinfiltrated with m03915-GFP or m04250-GFP to determine whether there was any NLS in the candidates, while GFP was control. The *N. benthamiana* leaves were collected at two days post infiltration (DPI) and were observed under an Olympus FV1000 MPE multiphoton laser scanning microscope (Tokyo, Japan).

### 4.5. Identification of host Proteins Interacting with m03915 and m04250 by Yeast Two-Hybrid (Y2H)

The construction of the library used for Y2H screening was described previously [8]. The *Saccharomyces cerevisiae* strain Y187 and pGADT7-Rec (*Sma*I-linearized) were used to construct the library of HLB infected sweet orange ‘Valencia’. The library construction was as the manual of the “Mate & Plate” Library System (Clontech, Cat no. 630490). Vector pGBKT7 DNA-BD (Clontech, Cat. No. 630443) was used to construct the bait plasmids. Primers m03915 YF/m03915 YMR (eliminate the autoactivation activity of m03915) and m04250 YF/m04250 YR were used to amplify the DNA fragments for construction bait plasmids pGBKT7-m03915E20A and pGBKT7-m04250. *Saccharomyces cerevisiae* Y2HGold was transformed with the two bait plasmids. The library screening was performed according to Matchmaker Gold Yeast Two-Hybrid (Clontech, Cat. No. 630489). The annotation and accession number of the identified positive prey were retrieved from the National Center for Biotechnology Information (NCBI). 

## Figures and Tables

**Table 1:** The host proteins interacted with mature proteins of CLIBASIA_03915 and CLIBASIA_04250.

Bait	Access Number	Protein ID	Annotation	Isolates
m03915E20A	XM_006465843	XP_006465906.1	cytochrome P450 71A1-like	1
	XM_006467728	XP_006467791.1	transcription factor bHLH162	1
	XM_006441298	XP_006441361.1	glutamate decarboxylase 5	1
	XM_006452415	XP_006470441.1	cold and drought-regulated protein	1
	XM_006481033	XP_006481096.1	ubiquitin-activating enzyme E1 1-like	1
	XM_006474049	XP_006474112.1	EG45-like domain containing protein	2
m04250	XM_006474512	XP_006474575.1	suppressor of mec-8 and unc-52 protein homolog 2	1

Yeast two-hybrid (Y2H) was used to identify the citrus proteins that interacted with the mature proteins of CLIBASIA_03915 without autoactivation activity (m03918E20A, the second E was converted to A to eliminate the autoactivation activity of m03915) and CLIBASIA_04250 (m04250).

## Supplementary Materials

The following are available online at https://www.mdpi.com/1422-0067/21/4/1414/s1, Figure S1: Distribution pattern of free GFP, m03915-GFP, and m04250-GFP in the epidermic cells of *N. benthamiana*; Table S1: The primers used in this research.
