# Supplementary material for: Heterologous Expression of CLIBASIA_03915/CLIBASIA_04250 by Tobacco Mosaic Virus Resulted in Phloem Necrosis in the Senescent Leaves of Nicotiana benthamiana
Source: Int J Mol Sci. 2020 Feb 19;21(4):1414. doi: 10.3390/ijms21041414 (PMC7073121; doi:10.3390/ijms21041414)
Supplement: Supplementary file 1 [file ijms-21-01414-s001.pdf]

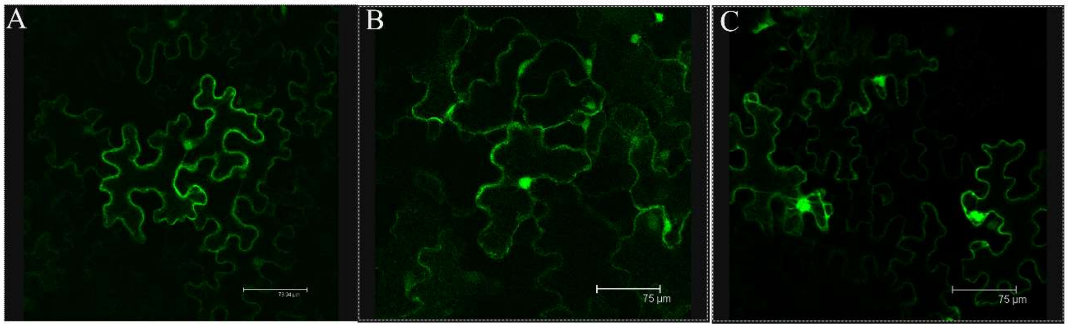

Figure S1. Distribution pattern of free GFP, m03915-GFP, and m04250-GFP in the epidermic cells of *N. benthamiana*. The full expanded leaves of six true leaf-stage *N. benthamiana* were used for agroinfiltration to determine the distribution patterns of free GFP, m03915-GFP, and m04250-GFP. Free GFP (A), m03915-GFP (B), and m04250-GFP showed the similar distribution pattern in the epidermic cells of *N. benthamiana*.

Table S1. Primers used in this research.

	**Primers used for phoA assay**
**CLIBASIA_03915 PF**	GAGCTCATGAATGCCAAGGGATTAATAGTAGC
CLIBASIA_03915 PR	CAATTGTTGTTCCTTTATTGCTATTTC
CLIBASIA_04250 PF	GAGCTCATGAATACAAGAATAATAGGAACCG
CLIBASIA_04250 PR	CAATTGTCTTCTGGATTGATTTTTACC
	Primers used for construction vectors for expression with TMV
m03915 EF	TTAATTAATGTGGAATCTAAAGCACGCAATTAGAAAG
m03915 ER	CTCGAGTTATTGTTCCTTTATTGCTATTTC
m03915 NF	TTAATTAATAATGGAATCTAAAGCACG
m04250 EF	TTAATTAATGTGTTCTTCTAAAAAAGGAGGTGAAAAAAAAG
m04250 ER	CTCGAGCTATCTTCTGGATTGATTTTTACCATTTTTC
m04250 NF	TTAATTAATGTTCTTCTAAAAAAGGAGGTGAAAAAAAAG
	Primers used for subcellular localization
GFP F	TCTAGAATGGCTAGCAAAGGAGAAGAACTTTTC
GFP R	GAGCTCTTATTTGTAAAGTTCATCCATGCCATGTGT
GFP FF	CCCGGGGCTAGCAAAGGAGAAGAACTTTTC
GFP FR	GAGCTCTTATTTGTAAAGTTCATCCATGCCATGTGT
m03915 GF	TCTAGATGTGGAATCTAAAGCACGCAATTAG
m03915 GR	CCCGGGTTGTTCCTTTATTGCTATTTC
m04250 GF	TCTAGATGTGTTCTTCTAAAAAAGGAGGTGAAAAAAAAG
m04250 GR	CCCGGGTCTTCTGGATTGATTTTTACCATTTTTC

HAint F	TCTAGATGTACCCATACGATGTTCCAGATTACGTAAGTTTCTGCTTCTACCTTTG
HAint R	GAGCTCCCCGGGGGATCCAGCCTGCACATCAACAAATTTTGGTCATATATTAG
RFP F	GGATCCGCCTCCTCCGAGGACGTCATCAAGG
RFP R	GAGCTCTTAGGCGCCGGTGGAGTGGCGGCCCTC
RFP-NLS F	GGATCCATGGCCTCCTCCGAGGACGTCATCAAGG
RFP-NLSR	GAGCTCTCTAGATCAGGTCGATCCCGACGCTATTTCCGAAGAATCAC
Primers used for yeast two-hybrid	
m03915 YF	GAATTCTGGAATCTAAAGCACGCAATTAG
m03915 YMR	GGATCCTTGGGCCTTTATTGCTATTTCTATCTTTC
m04250 YF	GAATTCTGTTCTTCTAAAAAAGGAGGTGAAAAAAAAG
m04250 YR	GGATCCCTATCTTCTGGATTGATTTTTACC

Underlines *indicate the added* restriction enzyme sites.
